# The Effect of Sub-MIC **β**-Lactam Antibiotic Exposure of *Pseudomonas aeruginosa* Strains from People with Cystic Fibrosis in a Desiccation Survival Model

**DOI:** 10.1155/2011/712618

**Published:** 2011-09-29

**Authors:** I. J. Clifton, M. Denton, F. M'Zali, D. G. Peckham

**Affiliations:** ^1^Regional Adult Cystic Fibrosis Unit, St James University Hospital, Leeds LS9 7TF, UK; ^2^Department of Microbiology, Leeds General Infirmary, Leeds LS1 3EX, UK

## Abstract

Prior to modern typing methods, cross-infection of *P. aeruginosa* between people with cystic fibrosis (CF) was felt to be rare. Recently a number of studies have demonstrated the presence of clonal strains of *P. aeruginosa* infecting people with CF. The aim of this study was to determine whether strains of *P. aeruginosa* demonstrated differences in resistance to desiccation and whether preincubation in subminimum inhibitory concentrations (MICs) of **β**-lactam affected desiccation resistance. The experimental data were modelled to a first-order decay model and a Weibull decay model using least squares nonlinear regression. The Weibull model was the preferred model for the desiccation survival. The presence of a mucoid phenotype promoted desiccation survival. Preincubation with antibiotics did not have a consistent effect on the strains of *P. aeruginosa*. Meropenem reduced desiccation resistance, whereas ceftazidime had much less effect on the strains studied.

## 1. Introduction

*Pseudomonas aeruginosa* is a gram-negative, nonfermentative, aerobic bacillus belonging to the family Pseudomonadaceae. The organism is ubiquitous within the environment and is particularly isolated from moist areas such as water and soil. *P. aeruginosa* causes chronic respiratory infections in people with cystic fibrosis (CF) and acts as opportunistic pathogen causing bacteraemia, urinary tract infections, and hospital acquired pneumonia in patients with burns, urinary catheters, and those on invasive ventilation [1]. Although* P. aeruginosa* is a nonfermentative aerobe, it can grow under anaerobic conditions using nitrate as an electron receptor. Its ability to survive in a wide range of environmental conditions is partially explained by its versatile nutritional abilities and its ability to resist high concentrations of salt, dyes, disinfectants, and many common antibiotics. The bacteria has been commonly found in the drains of wash basins in hospital wards [2], and aerosols containing *P. aeruginosa* can be detected when opening a tap [3, 4]. Isolation of *P. aeruginosa* from tap water is due to contamination of the tap itself, rather than the mains water supply [5].

Prior to the advent of modern genetic typing methods cross-infection of *P. aeruginosa* between people with CF was felt to be a rare event. More recently a number of studies have been undertaken that demonstrate the presence of clonal strains of *P. aeruginosa* infecting multiple patients in CF clinics [6–9].

*P. aeruginosa* is intrinsically resistant to most commonly used antibiotics. Antibiotic resistance is achieved through a combination of restricted antibiotic uptake through the outer membrane and a variety of energy-dependent mechanisms. The energy-dependent mechanisms through which *P. aeruginosa* achieves antibiotic resistance include efflux pumps and *β* lactamase-production. The energy-dependent mechanisms are usually under close regulation, and antibiotic resistance is often a result of mutations in the regulatory genes of these mechanisms [10]. Preincubation with antibiotics has been demonstrated to have a number of effects on *P. aeruginosa* including induction of a biofilm form of growth [11], improved heat and osmotic stress response [12], changes to hydrophobicity [13], and reduced bacterial adherence [14]. 

The aim of this study was to determine whether clonal strains of *P. aeruginosa*, identified as part of routine clinical sampling, demonstrated differences in resistance to desiccation and whether preincubation in subminimum inhibitory concentrations (MICs) of *β*-lactam antibiotics had an effect on the ability of the bacteria to resist desiccation. Both ceftazidime and meropenem are anti-pseudomonal antibiotics that are commonly used in the care of people with CF.

## 2. Materials and Methods

### 2.1. Bacterial Strains

The environmental strain of *P. aeruginosa* (NCIMB 10848) was obtained from the National Collection of Industrial and Marine Bacteria. Mucoid and nonmucoid variants of the Liverpool strain [15] and a nonmucoid variant of the Manchester strain [16] were obtained from the Centre for Infectious Disease, University of Edinburgh. All the other *P. aeruginosa *strains were obtained from clinical samples of sputa from people with CF and were genotyped by the Microbiology Department of Leeds General Infirmary using Pulsed-Field Gel Electrophoresis (PFGE) (See Table 1).

### 2.2. Antimicrobial Sensitivity Testing

MICs of ceftazidime and meropenem were performed on Iso-Sensitest (ISA) agar by Etest (AB Biodisk, Sweden) according to the manufacturer's guidelines [17]. MICs were read after 24 h incubation at 37°C.

### 2.3. Desiccation Survival Assay

#### 2.3.1. Preparation of Controlled Relative Humidity Chamber

Controlled conditions of relative humidity were established and maintained by the presence of saturated salt solution in an air tight plastic box. 30 ± 3% relative humidity (RH) was maintained by the presence of saturated solution of CaCl_2_·6H_2_O (Sigma Chemicals, UK) [19, 20]. The temperature of the room during the experiment was 22 ± 2°C. A digital thermohygrometer (Extech Instruments, USA) was used to monitor relative humidity and temperature.

#### 2.3.2. Inoculation of Glass Coverslips with Bacteria

After determination of the viable cell concentration curve, strains were grown to their maximum stationary phase cell concentration, and these cultures were used in the subsequent experiments for determining the survival on a dry surface. Strains were also grown for 24–48 h in nutrient broth or nutrient broth containing 0.25 × MIC concentrations of ceftazidime or meropenem at 37°C in air, with shaking at 110 rpm. 

A 1.0 mL aliquot of the nutrient broth culture was placed in a 1.5 mL Eppendorf tube and centrifuged for five minutes in a microcentrifuge (Eppendorf Centrifuge 5140) at 13000 g. The supernatant fluid was discarded and the cell pellet resuspended in 1.0 mL of distilled water. 20 *μ*L of the cell suspensions was deposited onto the sterile coverslips, and they were then placed in the controlled relative humidity chamber.

#### 2.3.3. Determination of Viable Count

Each glass coverslip was placed in 2 mL of sterile distilled water under aseptic conditions using a sterile pair of forceps. Following appropriate serial dilutions using sterile distilled water the cell suspension was inoculated onto Columbia blood agar plates using the spread plate method. After overnight incubation in air at 37°C, the number of colony forming units was counted using a colony counter. Three glass coverslips were used separately for each count and three different dilutions were made for each coverslip. Viable counts were determined at time 0 h, 1 h, 6 h, and 24 h.

### 2.4. Mathematical Models of Bacterial Inactivation

#### 2.4.1. First-Order Decay Model

The first-order decay model assumes that all the bacterial cells have an equal resistance to lethal treatment. This results in a linear relationship between the logarithm of the number of survivors and the treatment time, as described in the following first-order decay kinetics equation:

(1) $$\log\;\left(N_{t}\right)=\log\;\left(N_{o}\right)-kt,$$

where *N*_0_ = concentration at time 0, *N*_*t*_ = concentration at time *t*, *k* = inactivation rate, and *t* = time. 

#### 2.4.2. Weibull Model

The Weibull model of bacterial decay is a nonlinear model. It assumes that lethal events are probabilities and that the corresponding survival curves are cumulative forms of a distribution of lethal event, as described in (2) [21]. The shape of the survival curve is determined by *p*; when *p* < 1, the curve has a concave upwards appearance, when *p* > 1, the curve has a concave downwards appearance, and when *p* = 1, the survival curve is linear. The value *δ* represents the time to the first decimal reduction [22]. The scale and shape parameters are not independent; therefore an error in *δ* will be balanced by an error in *p*. Comparisons between survival curves were undertaken by comparing the value for *δ* with a fixed value for the shape parameter determined from the mean of the initial values for *p* [22]. See the following Weibull distribution equation [21]:

(2) $$\log\;\left(N_{t}\right)=\log\;\left(N_{0}\right)-{\left(\frac{t}{\delta }\right)}^{p},$$

where *N*_0_ = concentration at time 0, *N*_*t*_ = concentration at time *t*, *t* = time, *δ* = scale parameter, and *p* = shape parameter.

### 2.5. Statistical Analysis

The two models of bacterial decay were modelled to the experimental data by least squares error analysis using GraphPad Prism (GraphPad Inc, San Deigo, USA). Comparisons between curves were made using the *F*-Test. A *P* value of <0.05 was deemed significant.

Comparisons between Weibull survival curves were made with a fixed value for the shape parameter *p* determined by the mean of the values *p* for the strains studied.

## 3. Results

**Figure 1:**
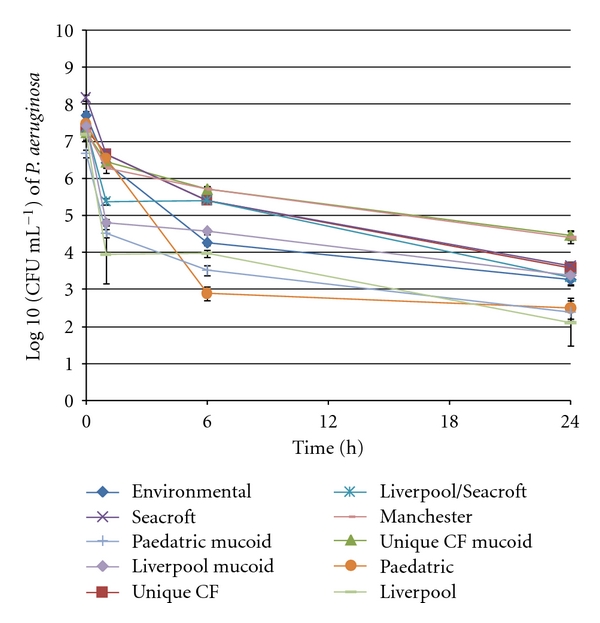
Mean viable counts of different strains of *P. aeruginosa* within the desiccation survival model. Error bars represent standard error of mean.

Within the first hour following inoculation of all the different strains of *P. aeruginosa* onto a dry glass surface, there was a rapid fall in viable counts of bacteria. All strains of *P. aeruginosa* were still able to be recovered at 24 hours but only at very low counts (Figure 1). 

### 3.1. Comparison of Mathematical Survival Curves Models in the Desiccation Model

The Weibull survival distribution was the preferred model for all the strains of *P. aeruginosa* examined in the desiccation survival assay (See Table 2). When the Weibull distribution model was applied to the experimental data all the strains of *P. aeruginosa* examined had a concave survival curve with *p* < 1 (See Table 2). The average value for the scale parameter *p* was determined to be 0.313.

### 3.2. Influence of Mucoid Phenotype on Desiccation Survival

**Figure 2:**
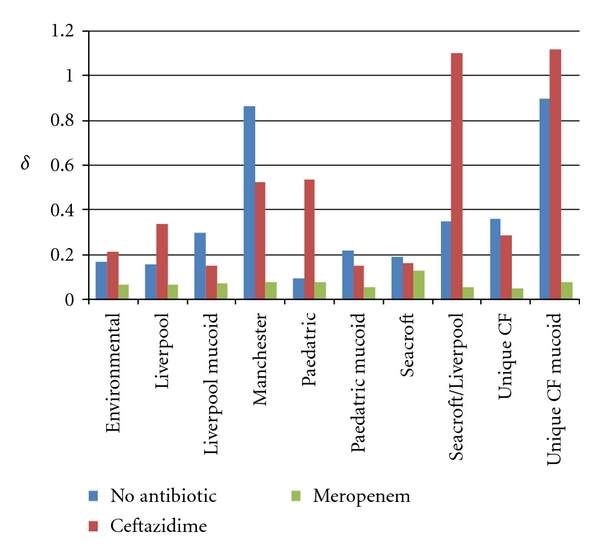
Values for *δ* determined from the Weibull distribution using a fixed value for the scale parameter *p* with preincubation with no antibiotic (*δ*), ceftazidime (*δ*_cef_), and meropenem (*δ*_mero_).

The value of *δ* was greater for all the mucoid strains of *P. aeruginosa* than the corresponding nonmucoid strain. This difference reached statistical significance for the Unique CF (*P* = 0.006) and Paediatric strains (*P* = 0.0476) (See Figure 2).

### 3.3. Influence of Antibiotic Preincubation on Desiccation Survival on Time to First Decimal Reduction

Preincubation with ceftazidime did not have any significant effect on any of the nonepidemic strains of *P. aeruginosa.* For epidemic strains it significantly increased the time to first decimal reduction for the Paediatric nonmucoid strain and the Seacroft/Liverpool strain (Paediatric non-mucoid *P* < 0.0001; Seacroft/Liverpool *P* = 0.0002) and significantly reduced the time to first decimal reduction Liverpool mucoid strain (*P* = 0.0143). The other epidemic strains were not significantly affected. 

All nonepidemic strains had a significant reduction in *δ* following preincubation with meropenem (Environmental *P* = 0.0002; Unique CF *P* < 0.0001; Unique CF Mucoid *P* < 0.0001). All epidemic strains of *P. aeruginosa* also had a significant reduction in *δ* following preincubation with meropenem, apart from the Paediatric, Seacroft, and Liverpool strains where the reduction in *δ* was not statistically significant (Liverpool mucoid *P* < 0.0001; Manchester *P* < 0.0001; Paediatric mucoid *P* < 0.0001; Seacroft/Liverpool *P* = 0.0007).

## 4. Discussion

There have been a number of studies demonstrating *P. aeruginosa* cross-infection between patients with cystic fibrosis [6, 7, 9, 23, 24]. The method of cross-infection is not clear. Dry surface contamination and aerosol dispersion have both been postulated as potential routes of transmission [15, 25]. An important factor that contributes to the loss of viability of bacteria both within aerosols and on dry surfaces is desiccation.

The use of the Weibull model to compare the survival curves of the different strains of *P. aeruginosa* allows for comparison of parameters of the survival curves and eliminates the impact of variations of the initial concentration of bacteria that may influence the time innocula may survive.

We have demonstrated that the mucoid phenotype is important for resistance to desiccation. All three strains available as both mucoid and nonmucoid phenotypes demonstrated greater resistance to desiccation when expressing the mucoid phenotype. This improved resistance to desiccation may be due to the alginate coating reducing the rate of evaporation of water from the bacteria, hence improving the ability of the organism to survive. Panagea et al. demonstrated no difference in survival of the Liverpool epidemic strain of *P. aeruginosa* regardless of the expression of a mucoid or nonmucoid phenotype [15]. Comparing an alginate deficient mutant of PAO1 with wild type, Chang et al. demonstrated that alginate production promoted desiccation resistance which would support the findings presented in the current study [26]. Skaliy and Eagon demonstrated that the *P. aeruginosa* cells in exponential phase of growth were most susceptible to the effects of desiccation compared to those in the stationary phase and that the addition of extracellular slime did not improve the desiccation resistance of exponential growth bacterial cells [27]. This would suggest that the high rate of metabolic activity associated with exponential growth may be more important to desiccation resistance than the presence of extracellular slime. 

There was no pattern of improved desiccation resistance between the epidemic strains and nonepidemic strains of *P. aeruginosa*. All three groupings of strains according to the value of *δ* contained both epidemic and nonepidemic strains of *P. aeruginosa*. These data are contrary to the data presented by Panagea et al [15]. They demonstrated that the Liverpool epidemic strain demonstrated prolonged survival compared to other strains. One explanation for the differences between the previous and current study would be the lack of control of relative humidity in the study of Panagea et al. [15]. 

A consistent effect of preincubation with meropenem was to reduce desiccation resistance in most of the strains studied. Carbapenems at sub-MIC levels have been demonstrated to decrease outer membrane permeability, improve heat and osmotic stress responses, and increase bacterial susceptibility to neutrophil phagocytosis [12].

Ceftazidime had the lesser effect on the strains studied, causing a significant change in survival in only three of the bacterial strains studied. While cephalosporins have been shown to reduce bacterial adherence to pneumonocytes and polymorphonuclear phagocytosis, they do not appear to affect the hydrophobicity of the bacterial surface [13, 14].

Other *β*-lactam antibiotics have also been demonstrated to modify the survival characteristics of *P. aeruginosa*. Preincubation with piperacillin-tazobactam has been shown to decrease adhesion, reduce motility, reduce twitching, reduce biofilm formation, and increase the sensitivity to oxidative stress [28]. 

This study demonstrated that *P. aeruginosa* can survive within a dry environment for prolonged periods of time and that the mucoid phenotype is an important factor promoting survival. It also demonstrates that preincubation with sub-MIC levels may have important effects on the physiology of the bacteria in relation to their resistance to desiccation. Promoting bacterial survival through antibiotic exposure could have important clinical consequences by potentiating the risk of cross infection between people with CF. Further studies should be undertaken looking at the role sub-MIC concentrations of different antibiotics may have in promoting or inhibiting cross-infection with epidemic strains of *P. aeruginosa* in CF units.

## Figures and Tables

**Table 1:** Details of *Pseudomonas aeruginosa* strains.

*Pseudomonas aeruginosa* strain		MIC (*μ*g mL^−1^)	
		Ceftazidime	Meropenem
Environmental strain	NCIMB 10848	1.0	0.32
Unique CF	4412061	256	32
Unique CF mucoid	4390364-1	2.0	0.50
Manchester [7]	2003/492	256	32
Liverpool/Seacroft	4390416-2	8	32
Seacroft	4390195	256	32
Liverpool [18]	2003/493	2.0	3.0
Liverpool mucoid [18]		8	0.5
Leeds Paediatric [6]	4410030	256	32
Leeds Paediatric mucoid [6]	7175611-1	1.5	32

**Table 2:** Comparison of first order decay and Weibull survival models.

	Model						
Bacterial strain	First order decay		Weibull			Preferred model	*P*
	*k*	*R* ^2^	*δ*	*p*	*R* ^2^		
Environmental	0.16	0.6599	0.15	0.30	0.9397	Weibull	<0.0001
Liverpool	0.03	0.4678	0.003	0.18	0.6918	Weibull	0.0001
Liverpool mucoid	0.12	0.5336	0.003	0.15	0.9618	Weibull	<0.0001
Manchester	0.10	0.8418	1.46	0.37	0.9478	Weibull	<0.0001
Paediatric	0.19	0.6104	0.16	0.34	0.8550	Weibull	<0.0001
Paediatric mucoid	0.14	0.5564	0.03	0.22	0.9635	Weibull	<0.0001
Seacroft	0.02	0.8168	0.31	0.35	0.9836	Weibull	<0.0001
Seacroft/Liverpool	0.14	0.7438	0.32	0.30	0.9107	Weibull	<0.0001
Unique CF	0.15	0.9026	1.73	0.51	0.9746	Weibull	<0.0001
Unique CF mucoid	0.10	0.8527	2.03	0.41	0.9402	Weibull	<0.0001
